# PFSA D50-U Proton-Exchange Gel Membrane for Symmetric Supercapacitors

**DOI:** 10.3390/gels12030223

**Published:** 2026-03-10

**Authors:** Borislava Mladenova, Mariela Dimitrova, Gergana Ivanova, Ivan Radev, Antonia Stoyanova

**Affiliations:** 1 Institute of Electrochemistry and Energy Systems “Acad. Evgeni Budevski”, Bulgarian Academy of Sciences, Acad. Georgi Bonchev Str., Block 10, 1113 Sofia, Bulgariamariela.dimitrova@iees.bas.bg (M.D.);; 2 The Hydrogen and Fuel Cell Center, ZBT GmbH, Carl-Benz-Str. 201, 47057 Duisburg, Germany; 3 National Centre of Excellence Mechatronics and Clean Technologies, Bul. Kliment Ohridski 8, 1756 Sofia, Bulgaria

**Keywords:** PFSA D50-U, gel polymer electrolyte, proton-exchange membrane, supercapacitors, symmetric, ionic conductivity, activated carbon

## Abstract

Gel polymer electrolytes are key components in next-generation energy storage systems, particularly supercapacitors, due to their high ionic conductivity, mechanical robustness, and operational safety. Ionomer-based gels derived from perfluorosulfonic acid (PFSA) are particularly promising, as their nanophase-segregated morphology enables the formation of three-dimensional ionic clusters capable of absorbing and retaining aqueous electrolytes. In this study, the commercial PFSA D50-U (Thasar S.r.l.) membrane was investigated for the first time as a gel-state ionomer electrolyte and separator in symmetric supercapacitors using coconut shell-derived activated carbon (YP-80F Kuraray Co., Ltd.). The effects of cation type on gel swelling, ionic conductivity, and electrochemical performance were investigated using Na_2_SO_4_ and Li_2_SO_4_ aqueous electrolytes. The results showed that PFSA D50-U formed stable gel structures, resulting in low internal resistance, high specific capacitance, and excellent long-term cycling stability. These findings demonstrate that PFSA D50-U is a novel proton-exchange gel membrane with strong potential for high-performance symmetric supercapacitors and other gel-based energy storage devices.

## 1. Introduction

The continuous growth in global energy consumption has significantly increased the demand for efficient, sustainable, and environmentally friendly energy storage systems. The development of technologies that combine high energy and power density with long service life and reduced environmental impact is therefore crucial for decreasing reliance on fossil fuels and facilitating the large-scale integration of renewable energy sources [1,2,3].

Supercapacitors are a class of electrochemical energy storage devices characterized by high power density, fast charge–discharge kinetics, and excellent cycling stability. These properties render them particularly suitable for applications necessitating the effective management of short-term energy fluctuations and for hybrid energy storage systems. The electrochemical performance of supercapacitors is strongly influenced by the properties of their key components, including electrodes, electrolytes, and separators. Collectively, these components determine ion transport, internal resistance, and overall device efficiency [4,5,6,7,8,9].

The separator fulfills a pivotal function by acting as an electrical insulation barrier between the electrodes, thereby facilitating efficient ion transport. The structural, porous, and chemical properties of the materials have been demonstrated to have a substantial impact on the ionic conductivity and long-term stability of supercapacitors. Research has shown that high porosity in ultra-thin separator membranes enables ion diffusion, reduces internal resistance, and enhances electrochemical performance, particularly at elevated current densities [10]. However, conventional separator materials frequently exhibit deficient ionic conductivity or inadequate durability, underscoring the necessity for advanced membrane-based solutions.

The ion-exchange membranes used in supercapacitors can be divided into two main categories: conventional single-layer membranes, which selectively facilitate the transport of specific ions (cations or anions), and bipolar membranes, which consist of two layers with opposing ion selectivities and can generate H^+^ and OH^−^ ions under the influence of an applied electric field [11]. Conventional cation-exchange membranes are composed of perfluorosulfonic acid (PFSA)-based materials, such as Nafion^®^ and Aquivion^®^, which are extensively used as proton-exchange membranes due to their high ionic conductivity and exceptional chemical stability. Their microphase-separated structure consists of hydrophilic gel domains with fixed sulfonic acid groups containing mobile ions, together with interconnected pores filled with an electrolyte solution, which ensures efficient ion transport and charge balance [12,13,14,15,16,17,18,19,20]. Notwithstanding these advantages, the high cost and limited availability of these membranes constrain their extensive implementation in scalable energy storage devices.

PFSA D50-U is based on a chemically (end-group) stabilized, long-side chain PFSA with an ion-exchange capacity of 1.02 meq g^−1^ (EW = 980 g mol^−1^ SO_3_H). The membrane was originally developed for heavy duty proton-exchange membrane fuel cells (PEMFC). It is supplied as a solution-cast, unreinforced film (50 µm) and provides similar proton conductivity to Nafion 212 while being currently more accessible. To the best of our knowledge, PFSA D50-U has not yet been systematically evaluated in symmetric supercapacitors, which motivates the present study.

Concurrently, investigations have been conducted into the potential of alternative materials for use in separators and membranes. In addition to keratin-based biomaterials, cellulose separators with micro- and nanostructures have been developed, facilitating ion movement and improving electrochemical properties [21,22]. Synthetic polymer membranes, such as poly (vinyl alcohol) with high Mg^2+^ ion conductivity, also demonstrate stability and efficiency in supercapacitor applications, ensuring reliable ion transport and improved performance [23,24].

Despite substantial advancements witnessed in the domain of membrane supercapacitors, the utilization of commercial PFSA membranes in gel form as electrolytes and separators within symmetric supercapacitors remains to be examined. Additionally, the impact of cation type on the ionic conductivity, internal resistance, and electrochemical behavior of PFSA-based gel membranes remains to be systematically investigated. A comprehensive understanding of these effects is imperative for the optimization of ion transport mechanisms and the enhancement of device performance [25].

In this context, the present study evaluates the commercial PFSA D50-U membrane as a gel-state ion-exchange electrolyte and separator in symmetric supercapacitors based on coconut shell-derived activated carbon (YP-80F, Kuraray Co., Ltd.). A systematic investigation was carried out to determine the effect of cation type on ionic conductivity and overall electrochemical performance. Physicochemical characterization includes X-ray diffraction (XRD) and scanning electron microscopy with energy-dispersive X-ray spectroscopy (SEM/EDX). Electrochemical analysis is carried out using cyclic voltammetry, galvanostatic charge–discharge measurements, and electrochemical impedance spectroscopy. Particular emphasis is placed on internal resistance, specific capacitance, and long-term cycling stability.

## 2. Results and Discussion

### 2.1. Physicochemical Analysis of the Membrane

**Figure 1:**
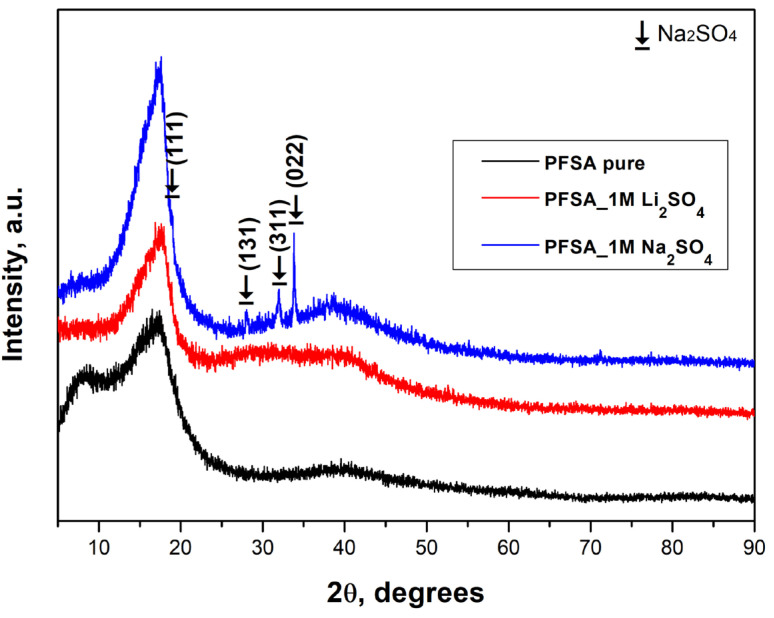
XRD patterns of pure and activated PFSA membranes. The marked reflections correspond to orthorhombic Na_2_SO_4_; (PDF 00-037-1465).

Figure 1 shows the XRD patterns of the pure and activated PFSA membranes. The diffractogram of the pure PFSA membrane displays a broad amorphous halo, within which weak structural features can be distinguished at 2θ ≈ 8–9° and 17–18°. A very low-intensity hill-like elevation is also observed around 2θ ≈ 39°. These broad maxima, originating from scattering by partially ordered polymer chains, indicate an ordered structure within the ion-exchange domains embedded in the amorphous polymer matrix. This confirms the semicrystalline structure typical of proton-conducting membranes such as PTFE and Nafion^®^, whose crystallinity usually ranges between 5 and 25% [26,27,28]. The influence of different electrolytes (Li_2_SO_4_ and Na_2_SO_4_) is clearly reflected in the corresponding diffractograms. Both activated membranes exhibit a narrowing and sharpening of the main peak, suggesting improved organization within the hydrophilic regions. This structural rearrangement is associated with an increase in the effective degree of crystallinity, which correlates with the enhanced water uptake of the ion-exchanged forms. In the case of activation with sodium sulfate, the appearance of additional (although low-intensity) diffraction features further support the stronger structural ordering. This effect can be attributed to the smaller hydrated radius of Na^+^ compared to Li^+^, which enables a more compact packing and stronger ionic cross-linking between the sulfonic acid groups. These observations are confirmed by the calculated crystallinity values (%χ), obtained through deconvolution of the amorphous halo and crystalline contributions. The crystallinity values as a function of the cationic form (Li^+^ or Na^+^) are summarized in Table 1.

An increase in structural ordering is indirectly associated with enhanced mechanical stability and reduced swelling, as widely reported for PFSA membranes with more compact and better-defined ionic domains [29]. Consequently, in our case, although no direct mechanical or swelling measurements were performed, the observed increase in crystallinity appears to fall within a range that allows for a favorable balance: the membrane maintains high proton conductivity while being structurally favorable for potentially enhanced mechanical strength, thermal stability, and swelling resistance, in agreement with established literature trends [29,30].

**Figure 2:**
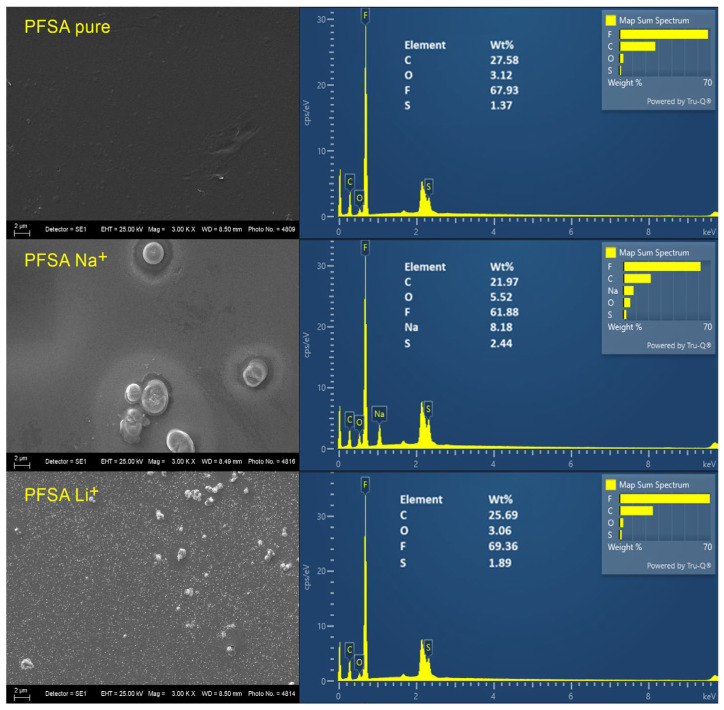
SEM–EDX analysis of the pure PFSA D-50U membrane and PFSA D-50U membranes activated with Na^+^ and Li^+^.

The surface morphology of the pure membrane and the treated membranes (Figure 2) is consistent with the structural features revealed by the XRD analysis. The pure PFSA D50-U membrane exhibits a smooth and uniform surface, characteristic of the unactivated polymer. After activation in Li_2_SO_4_, the membrane shows a fine and evenly distributed layer of lithium sulfate, indicating a homogeneous distribution of Li^+^ ions within the ionic domains.

In contrast, the membrane activated with Na_2_SO_4_ exhibits an uneven distribution of salt on its surface, forming discrete aggregates of sodium sulfate crystals. Each aggregate is surrounded by a distinct interfacial zone, indicating a localized rearrangement of the hydrophilic ionic domains. This rearrangement likely facilitates ion-mediated associations between sulfonate groups, leading to regions that are heterogeneous in both mechanical and ionic properties. As a result, the capacitive behavior may vary spatially across the membrane.

These morphological differences correlate directly with the X-ray diffraction results: the Li^+^-activated membrane exhibits moderate sharpening of the main amorphous peak, whereas the Na^+^-activated membrane shows peak narrowing along with additional low-intensity diffraction features. This confirms that Na^+^ induces more pronounced local ordering in the hydrophilic domains compared to Li^+^.

Elemental analysis (Figure 2) further confirms the presence of the constituent elements of the polymer matrix. In the membrane treated with Na^+^, sodium is detected. However, Li^+^ could not be observed due to the inherent detection limits of the EDX technique for light elements.

**Figure 3:**
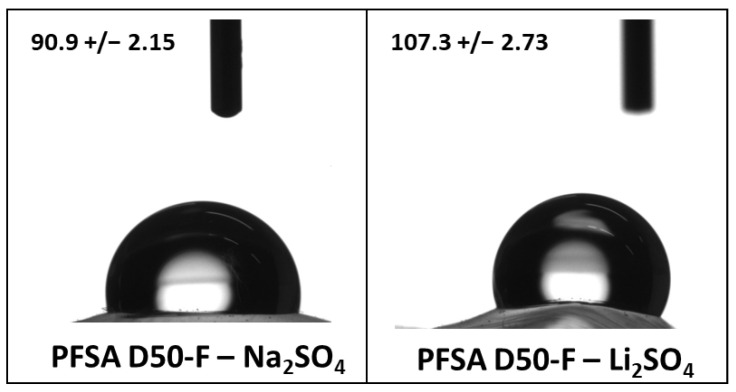
Wetting angles of the PFSA D50-F membrane with 1 M Na_2_SO_4_ and 1 M Li_2_SO_4_ electrolytes.

The wetting angles (see Figure 3) of the PFSA D50-U membrane in Na_2_SO_4_ and Li_2_SO_4_ were 90.9° ± 2.15° and 107.3° ± 2.73°, respectively. The lower contact angle in the Na_2_SO_4_ solution indicates improved interfacial wetting and reduced interfacial resistance, which is consistent with the higher capacitance observed at low current densities. At higher current densities, however, ion transport limitations become increasingly significant. In the case of Li_2_SO_4_, the specific transport characteristics of the ions and their interactions with the membrane may partially offset the larger contact angle, leading to more stable electrochemical behavior. Overall, these results suggest that wettability is the main factor governing performance at low current densities, whereas ion transport processes dominate at higher currents.

### 2.2. Electrochemical Analysis

**Figure 4:**
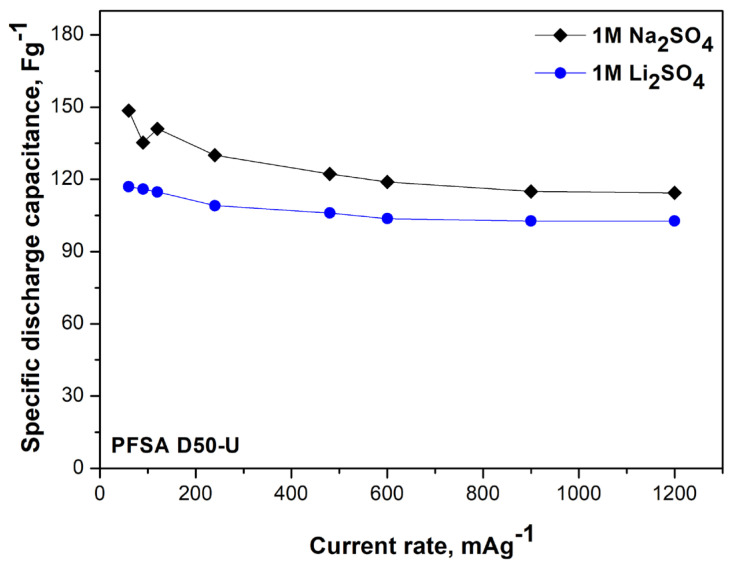
Specific discharge capacitance as a function of current load for symmetric supercapacitors with proton-exchange PFSA membranes for lithium- and sodium-ion systems.

The specific discharge capacitance of PFSA-based supercapacitors at varying current densities is shown in Figure 4. The investigation revealed that both the Li^+^- and Na^+^-based systems demonstrated high capacity values and relatively stable performance. The sodium-ion supercapacitor demonstrates a superior specific capacitance, ranging from approximately 150 F/g at low current loads to approximately 115 F/g at higher currents. There is a slight fluctuation at low currents that gradually attenuates as the current increases. Conversely, the lithium-based supercapacitor demonstrates a reduced specific capacitance, exhibiting a decline from approximately 116 F/g to 103 F/g with increasing current. However, it exhibits better stability across the entire current range. These results underscore the key role of the PFSA gel membrane in providing homogeneous ionic pathways, ensuring efficient charge storage for both electrolytes and accounting for the observed disparities in performance.

**Figure 5:**
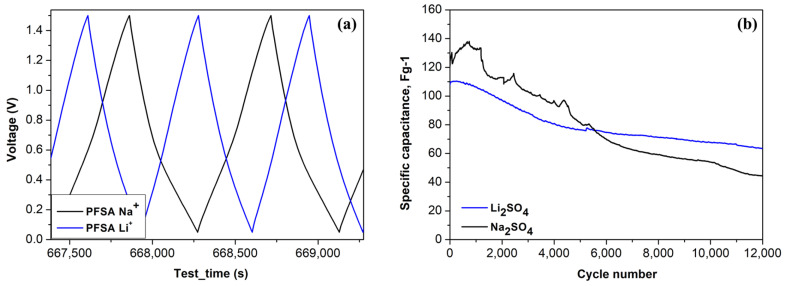
Galvanostatic charge–discharge curves (**a**) and long-term cycling (**b**) at 240 mA g^−1^ for symmetric PFSA-membrane supercapacitors with Li_2_SO_4_ and Na_2_SO_4_ electrolytes.

The galvanostatic charge–discharge (GCD) curves recorded at 240 mA g^−1^ (Figure 5a) substantiate these observations. The Li^+^-based supercapacitor demonstrates lower IR drop and remarkably symmetrical triangular charge–discharge profiles, suggesting reduced internal resistance and enhanced charge transport efficiency. Conversely, the Na^+^-based system, although operating within a comparable potential window, exhibits slightly elevated levels of asymmetry and prolonged charge–discharge periods, indicative of slower ion kinetics. The maintenance of the shape and amplitude of the curve over successive cycles indicates the efficacy of the PFSA gel electrolyte in preserving structural integrity and ionic connectivity, thereby demonstrating good reversibility and cycle stability in both systems. The nearly linear and symmetrical triangular profiles observed for both electrolytes are indicative of a predominantly electrostatic charge storage mechanism, consistent with EDLC-type behavior.

The device’s performance is contingent upon the ionic conductivity and ion distribution within the electrolyte–membrane system. In the context of a sodium sulfate electrolyte, the observation of larger sulfate aggregates on the proton-exchange membrane, frequently accompanied by halogen characteristics, indicates a less uniform distribution of ions. This heterogeneity likely contributes to the observed fluctuations in specific capacity, especially at low current densities. In contrast, the lithium electrolyte consists of smaller and more uniformly distributed ions, thereby facilitating more homogeneous ion migration through the membrane and more stable electrochemical behavior. Consequently, while the sodium-based supercapacitor demonstrates a higher specific capacitance, its performance is less stable over time. The lithium-based device, despite exhibiting a slightly lower specific capacitance, demonstrates superior stability and reproducibility during both short-term measurements and long-term cycling (Figure 5b). These trends indicate a trade-off between capacitance value and electrochemical stability, which is primarily governed by ion size, mobility, and distribution within the PFSA gel electrolyte. The sudden drop in the Na_2_SO_4_-based system is attributed to diffusion-controlled transport limitations rather than irreversible structural degradation. Na^+^ ions, with a larger hydrated radius and lower mobility than Li^+^, exhibit longer diffusion and relaxation times within the confined hydrophilic ionic domains of the PFSA membrane and the hierarchical carbon pore network. As cycling proceeds, concentration gradients and polarization effects intensify, particularly at higher current densities, reducing effective ion accessibility and causing a rapid apparent capacitance decline. This explanation is consistent with increased diffusion-related impedance in the EIS spectra (Table 2) and the rate-dependent behavior observed in cyclic voltammetry.

**Figure 6:**
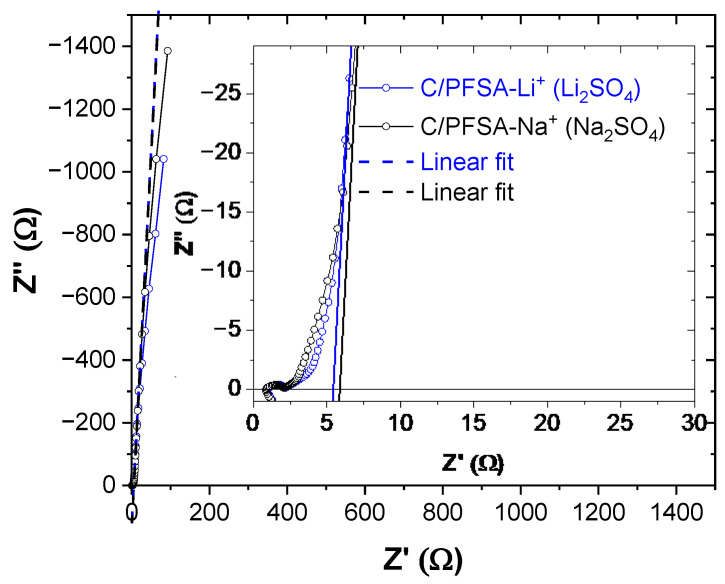
Nyquist impedance spectra of the symmetric PFSA-based supercapacitors with Li_2_SO_4_ and Na_2_SO_4_ electrolytes, recorded prior to galvanostatic cycling. Blue and black circles correspond to the measured impedance spectra for the Li_2_SO_4_ and Na_2_SO_4_ systems, respectively. The dashed lines represent linear fits applied to the low-frequency region of the spectra.

Electrochemical impedance spectroscopy (EIS) provides further insight into the electrochemical behavior of the symmetrical supercapacitors with PFSA membranes (Figure 6 and Table 2). Prior to the galvanostatic testing, Nyquist plots were recorded in the frequency range of 100 kHz to 1 mHz, thereby demonstrating the characteristic features of porous carbon/electrolyte systems. Reliable equivalent-circuit fitting was not possible due to the highly suppressed and overlapping semicircles and the elongated diffusion tails, which make the fitted parameters non-unique and increasingly unreliable when low frequencies are included [31]. Therefore, the spectra were analyzed directly from the Nyquist diagrams.

The high-frequency intercept of the semicircle with the Z′-axis (Z″ → 0) was taken as the series resistance Rs, reflecting the combined contributions of electrode electronic resistance, electrode/electrolyte contact resistance, and the ionic resistance of the PFSA membrane containing Li^+^ or Na^+^ ions. Slightly lower Rs values were observed for Na_2_SO_4_ (Table 2). The depressed semicircle at intermediate frequencies arises from the superposition of pore resistance Rp and charge-transfer resistance Rct of the two electrodes, with the right-hand intercept used to estimate Rs + Rp + Rct. The extracted Rp + Rct values are very similar for both electrolytes.

At low frequencies, the Nyquist plots develop steep diffusion-limited tails (~88°), indicative of finite-length Warburg behavior. Linear fitting of this region allows estimation of the internal resistance, Rint = Rs + Rp + Rct + Rd, where Rd represents diffusion-related resistance. Despite its slightly lower Rs, the Na^+^-based supercapacitor exhibits a higher overall Rint due to a larger Rd, attributed to the larger ionic radius and lower mobility of Na^+^ ions, which experience stronger interactions and steric hindrance within the PFSA/carbon pore network. In contrast, smaller Li^+^ ions migrate more efficiently, resulting in lower Rint and faster charge/discharge, though the total charge accumulation at low currents is slightly lower. This explains the trade-off between higher low-frequency capacitance for Na^+^ and lower internal resistance for Li^+^.

**Figure 7:**
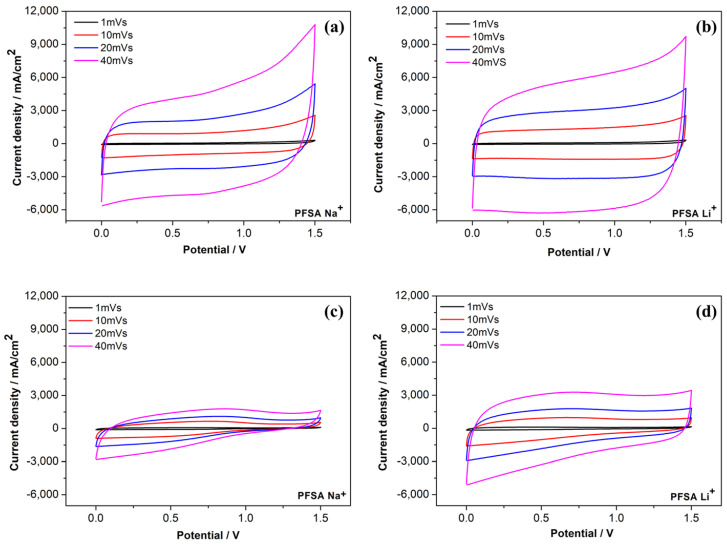
CV curves of symmetric supercapacitors with a proton-exchange PFSA membrane at different scan rates (1–40 mV/s). (**a**,**b**) Cells before cycling; (**c**,**d**) after 12,000 cycles. (**a**,**c**) 1 M Na_2_SO_4_; (**b**,**d**) 1 M Li_2_SO_4_.

Cyclic voltammetry (CV) measurements further support these observations (Figure 7). The quasi-rectangular CV profiles recorded at scan rates ranging from 1 to 40 mV s^−1^ for both 1 M Na_2_SO_4_ and 1 M Li_2_SO_4_ electrolytes confirm predominantly electrostatic (EDLC-type) charge storage, with no significant Faradaic contributions within the investigated potential window. The Li^+^-based system demonstrates higher current densities across the entire potential range, indicative of faster ion kinetics, lower internal resistance, and more efficient ion connectivity. This is consistent with the smaller IR drops and more symmetric GCD profiles observed in Figure 5a. Although the EIS reveals a slightly lower high-frequency ohmic resistance for Na_2_SO_4_-based cell, CV and GCD measurements probe the effective internal resistance under current operation, which additionally includes charge transfer and diffusion related contributions. In this regime, the higher mobility and smaller size of Li^+^ ions enable more homogeneous transport within the porous carbon network, reducing polarization during cycling. Conversely, the Na^+^-based supercapacitor demonstrates a more pronounced increase in current at higher potentials, suggesting stronger interactions between the ions and the electrode and diffusion-limited behavior. This is consistent with the longer charging and discharging times and capacity fluctuations observed during the cycles.

Furthermore, in the context of extended charge and discharge cycles, Na_2_SO_4_ demonstrates a tendency to undergo CV profile deformation and exhibit an increased capacitance loss (Figure 7c). Notwithstanding its diminished initial capacitance, Li_2_SO_4_ manifests a more stable and symmetrical CV profile with negligible capacity loss, thereby substantiating the retention of its electrochemical characteristics (Figure 7d). A comprehensive evaluation of the CV results reveals again a discernible trade-off between specific capacitance and electrochemical stability. While the sodium-based system can achieve higher capacitance values, the lithium-based electrolyte provides more homogeneous ion transport and better operational stability. This is due to differences in ion size, mobility, and interactions between the membrane and electrolyte.

**Figure 8:**
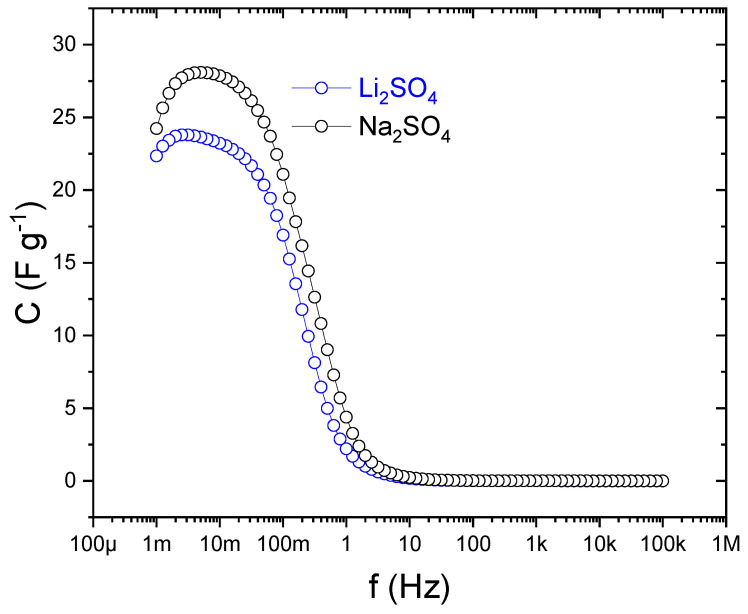
Frequency dependence of the specific capacitance Cs of symmetric supercapacitors employing PFSA membranes equilibrated with either Li_2_SO_4_ or Na_2_SO_4_ electrolytes before the galvanostatic measurements.

Frequency-dependent specific capacitance Cs(f) was derived from the complex-capacitance formalism applied to the EIS spectra (Figure 8):

The specific capacitance (Figure 8) was obtained from the complex-capacitance formalism applied to the impedance spectra,

(1) $$Cs\;\left(f\right)=-\frac{Z^{\prime \prime }}{2\pi f({Z^{\prime }}^{2}+{Z^{\prime \prime }}^{2})}$$

and normalized by the total active mass of both electrodes (0.0068 g C for Li_2_SO_4_ and 0.00472 g C for Na_2_SO_4_ cells). At high frequencies (10^4^–10^5^ Hz), *Cs* is limited by ohmic resistance and the inability of ions to follow rapid AC perturbations, whereas decreasing frequency enables progressively deeper pore regions to participate in charge storage, resulting in continuous increase in *Cs*. Below ~1 Hz, both systems exhibit a pronounced low-frequency enhancement characteristic of finite-length (open) Warburg behavior, reflecting constrained ion diffusion in the porous carbon network.

Both systems show similar qualitative trends, indicating comparable pore accessibility and interfacial kinetics in the PFSA matrix. Quantitatively, the Na_2_SO_4_-based cell exhibits slightly higher low-frequency capacitance, consistent with the lower mobility and larger hydrated radius of Na^+^, which enhance diffusion-controlled contributions. This trend agrees with the higher DC capacitance obtained from the galvanostatic charge–discharge measurements. It should be noted that the complex-capacitance formalism describes the dynamic AC response and does not represent the static DC capacitance of the supercapacitor.

## 3. Conclusions

In this study, PFSA D50-U has evaluated for the first time as a gel-state proton-exchange membrane in symmetric supercapacitors using both Li^+^ and Na^+^ electrolytes. The membrane demonstrates low internal resistance, high specific capacitance, and excellent cycling stability. Specifically, Li^+^ facilitates homogeneous ionic conduction and ensures operational stability, while Na^+^ offers higher capacitance at low currents but is constrained by diffusion limitations and exhibits greater performance. Structural and morphological analyses reveal stronger local ordering with Na^+^ and more uniform ionic domains with Li^+^. Frequency-dependent capacitance has been demonstrated to be a reliable indicator of comparable pore accessibility and interfacial kinetics. The presence of Na^+^ has been shown to provide a modest enhancement at low frequencies. A trade-off between capacitance and stability, governed by ion size, mobility, and membrane–electrolyte interactions, positions PFSA D50-U as a promising material for high-performance gel-based energy storage devices.

## 4. Materials and Methods

### 4.1. Materials

In the present work, commercial coconut shell-derived activated carbon YP-80 F (Kuraray Co., Ltd., Tokyo, Japan) was used for electrode fabrication. The electrodes were prepared Via the casting method, with a slurry composed of 80 wt.% YP-80F, 10 wt.% ABG 1005 EG1 graphite, and 10 wt.% polyvinylidene fluoride (PVDF) dissolved in 1-methyl-2-pyrrolidone. After coating onto a glass plate, the electrodes were dried and thermally treated in several steps to remove residual solvent and enhance their stability, as described in our previous work [32]. A commercial proton-exchange membrane PFSA D50-U (Thasar S.r.l., Milan, Italy) was employed as both the separator and the solid-state electrolyte. Prior to use, the membrane was immersed in distilled water to remove the protective layer and subsequently conditioned in the corresponding electrolyte solution (1 M Na_2_SO_4_ or 1 M Li_2_SO_4_) for 24 h to ensure proper ion exchange and stabilization. Electrochemical measurements were conducted to evaluate the membrane performance in electrochemical energy storage devices such as supercapacitors.

### 4.2. Physicochemical Characterization of Materials

The proton-exchange membranes were investigated using X-ray diffraction (XRD) and scanning electron microscopy with energy-dispersive X-ray spectroscopy (SEM-EDX). Structural analysis was conducted on a PANalytical Aeris X-ray powder diffractometer (Malvern Panalytical, Almelo, The Netherlands) with Cu Kα radiation (λ = 1.5406 Å) in a θ–θ Bragg–Brentano configuration, recording diffraction patterns over a 2θ range of 5–90° with a step size of 0.02° and a counting time of 60 s per step. Phase identification and diffractogram analysis were performed using HighScore Plus 5.1 software and the ICDD PDF-2 (2022) database. Morphological and elemental analyses were carried out using a Zeiss Evo 10 SEM (Carl Zeiss Microscopy, Oberkochen, Germany) equipped with an Oxford Ultim Max 40 EDX detector (Oxford Instruments, Abingdon, United Kingdom). SEM operation was performed using Smart SEM software (V07.05), and the EDX results were processed with AZtec software (version 6.1 HF4). Both the pure membrane and those treated with sodium (Na^+^) or lithium (Li^+^) ions were examined to evaluate surface features and cation distribution within the membrane matrix. The contact angle was determined by depositing a 10 μL droplet of each electrolyte (Na_2_SO_4_ and Li_2_SO_4_) onto the membrane surface. All tensiometric measurements were performed using a KRÜSS K100 (KRÜSS GmbH, Hamburg, Germany) force tensiometer.

### 4.3. Electrochemical Characterization

The electrochemical behavior of the solid-state supercapacitors was systematically investigated using several complementary techniques. Galvanostatic charge–discharge (GCD) measurements, along with cycling stability tests, were carried out on an Arbin BT-2000 battery testing system. The GCD experiments were performed within a voltage window of 0.05–1.6 V, applying current rates ranging from 60 to 1200 mA g^−1^, with 30 consecutive cycles recorded at each current step. Cycling durability was assessed by subjecting the devices to continuous charge–discharge operation at a constant current density of 240 mA g^−1^ for up to 12,000 cycles. Cyclic voltammetry (CV) measurements were conducted using a Multi PalmSens potentiostat (PalmSens 4, Houter, The Netherlands) at a fixed voltage window and scan rates of 1, 10, 20, and 40 mV s^−1^. Electrochemical impedance spectroscopy (EIS) was performed using the same instrument over a frequency range extending from 10 MHz down to 1 mHz. The specific discharge capacitance (F g^−1^) was determined from the galvanostatic charge–discharge profiles using Equation (2):

C = (I × Δt)/(m × ΔV), (2)

where I (A) is the applied discharge current, Δt (s) represents the discharge time, m (g) is the mass of the electrochemically active material, and ΔV (V) denotes the operating voltage window.

## Figures and Tables

**Table 1:** Degree of crystallinity (χ%) of PFSA membranes.

Sample	χ [%]
PFSA pure	17.71
PFSA/Li^+^	24.13
PFSA/Na^+^	37.32

**Table 2:** Resistance contributions extracted from the impedance spectra.

	Rs [mΩ]	Rs + Rp + Rct [mΩ]	Rp + Rct [mΩ]	Rint [mΩ]	Rd [mΩ]
Li_2_SO_4_	963	2195	1232	5435	3240
Na_2_SO_4_	877	2080	1203	5878	3798

## Data Availability

The data that support the findings of this study are available within the articles.
